# Comparing health outcomes between coronary interventions in frail patients aged 75 years or older with acute coronary syndrome: a systematic review

**DOI:** 10.1007/s41999-022-00667-9

**Published:** 2022-07-31

**Authors:** Gregory W. van Wyk, Shlomo Berkovsky, David Fraile Navarro, Enrico Coiera

**Affiliations:** Australian Institute of Health Innovation, Faculty of Medicine, Health and Human Sciences, Macquarie University, 75 Talavera Rd, Macquarie Park, NSW 2113 Australia

**Keywords:** Acute coronary syndrome, Frailty, 75 years or older, Angiography, Percutaneous coronary intervention, Coronary artery bypass grafting

## Abstract

**Purpose:**

To assess current evidence comparing the impact of available coronary interventions in frail patients aged 75 years or older with different subtypes of acute coronary syndrome (ACS) on health outcomes.

**Methods:**

Scopus, Embase and PubMed were systematically searched in May 2022 for studies comparing outcomes between coronary interventions in frail older patients with ACS. Studies were excluded if they provided no objective assessment of frailty during the index admission, under-represented patients aged 75 years or older, or included patients with non-ACS coronary disease without presenting results for the ACS subgroup. Following data extraction from the included studies, a qualitative synthesis of results was undertaken.

**Results:**

Nine studies met all eligibility criteria. All eligible studies were observational. Substantial heterogeneity was observed across study designs regarding ACS subtypes included, frailty assessments used, coronary interventions compared, and outcomes studied. All studies were assessed to be at high risk of bias. Notably, adjustment for confounders was limited or not adequately reported in all studies. The comparative assessment suggested a possible efficacy signal for invasive treatment relative to conservative treatment but possibly at the risk of increased bleeding events.

**Conclusions:**

There is a paucity of evidence comparing health outcomes between different coronary interventions in frail patients aged 75 years or older with ACS. Available evidence is at high risk of bias. Given the growing importance of ACS in frail patients aged 75 years or older, new studies are needed to inform optimal ACS care for this population. Future studies should rigorously adjust for confounders.

## Key Summary Points

**Aim:**

To assess the current evidence comparing the health outcomes of coronary interventions in frail patients aged 75 years or older with acute coronary syndrome.

**Findings:**

Available studies are observational and limited by incomplete statistical adjustment required for robust causal analysis. There may be a signal for improved outcomes in acute coronary syndrome patients treated invasively vs conservatively.

**Message:**

Robust studies are needed to inform the optimal selection of coronary interventions in frail older patients with acute coronary syndrome.

## Introduction

Acute coronary syndrome (ACS) is the emergency manifestation of coronary heart disease, the leading cause of death globally [1–3]. The relative contribution of ACS to mortality increases with age, and the absolute number of annual deaths in people aged 75 years or older is far higher than that in people younger than 75 years [1–4]. As the population ages, the contribution of the people aged 75 years or older to the ACS case mix is expected to rise. Correspondingly, as ageing is strongly associated with increasing frailty risk, frailty is likely to be an increasingly common complicating factor [5]. Frailty complicates clinical care, because it is associated with poor outcomes and increases the risk of a range of adverse effects from procedures and pharmacological treatments [6, 7]. Procedures central to ACS management are angiography and reperfusion procedures, including thrombolysis, percutaneous coronary intervention (PCI) and coronary artery bypass grafting (CABG) [6, 8]. Therefore, determining which coronary interventions (i.e., strategies and reperfusion procedures) optimize outcomes in frail older people with ACS is a matter of significant public health importance [9]. To assess the current evidence comparing the health outcomes of available coronary interventions in frail patients aged 75 years or older with different subtypes of ACS, we conducted a systematic review of the literature.

## Methods

The methods employed in the study adhered to the preferred reporting items for systematic reviews and meta-analyses (PRISMA) recommendations [10].

### Search strategy and selection criteria

Scopus, Embase and PubMed were searched in May 2022 for English records reported since January 1990, and retrieved records were imported into EndNote X9 for de-duplication, screening, and eligibility determination [10, 11]. Next, the title and abstract of unique records were screened against inclusion and exclusion criteria. Full-text articles for the remaining records were retrieved and reviewed to determine eligibility.

To review the impact of different coronary interventions in frail patients aged 75 years or older across the range of ACS presentations, all coronary interventions and ACS presentations were included in the search. In addition, any method for categorizing frailty was permitted, providing an analysis of outcomes in patients categorized as frail was included in the publication. An informal review of key ACS and frailty guidelines informed the search terms used in the search strategy [6, 8, 12, 13]. The complete set of inclusion and exclusion criteria and specific search terms employed in the search is detailed in Table 1. The search strings used for each database search are provided in the appendix (Table 5).

**Table 1:** Search terms used to identify articles for review

PICO	Inclusion criteria	Search terms	Exclusion criteria
Population and	Frail, and	"frail" OR "multimorbid" OR "highly comorbid"	No assessment of frailty during the index admission
	Elderly, and	"elderly" OR "older" OR "old"	If the study includes patients aged ≤ 75 and no subgroup analysis is presented for patients aged ≥ 75, or if the mean/median age < 75
	Any ACS	"acs" OR "acute coronary" OR "myocardial infarction" OR "unstable angina" OR" "stemi" OR "nstemi" OR "nsteacs" OR "nste-acs" OR "ua"	If the study includes non-ACS patients, e.g., stable angina, and no subgroup analysis is presented for ACS subgroup
Interventions, and	Any coronary intervention strategy or reperfusion treatment (including revascularisation procedures)	"pci" OR "percutaneous coronary intervention" OR "angiogra*" OR "invasive management" OR "invasive strategy" OR "medical management" or "conservative strategy" OR "conservative management" OR "cabg" OR "coronary artery bypass" OR "thromboly*"	
Comparisons, and	Pairwise comparison of outcomes between any two coronary interventions from a Randomized controlled trial (RCT), a Meta-analysis or an observational study	"treatment effect" OR "treatment benefit" OR "treatment outcomes" OR "versus" OR "vs" OR "compar*"	No comparative outcomes between treatments are presented, e.g., a methods paper Not a primary research article, e.g., a review article
Outcomes	Any health outcome. Examples include all-cause death, recurrent myocardial infarction, stroke, rehospitalization, quality of life and bleeding	No limits applied	
Filters		English, from 1990 to latest	

### Data items, synthesis methods and risk of bias assessments

Data from each article were extracted and tabulated using the following set of pre-specified characteristics:

- Study design, e.g., RCT, observational study.
- Data sources, e.g., registry, administrative data set.
- Population characteristics including age, ACS subclass, frailty scale (score, index), and the number of frail patients.
- Interventions compared, e.g., invasive vs conservative strategy and PCI vs CABG.
- Treatment outcomes, including primary outcome measures and results.

We conducted a qualitative synthesis of results, including an overview of the studies' design characteristics and results and a risk of bias assessment. Two reviewers (GvW & DFN) conducted the risk of bias assessment using McMaster’s CLARITY group Tool for Assessing Risk of Bias in observational studies [14]. After reviewing the studies independently, the reviewers discussed their findings to reach a consensus and, with the help of a third reviewer (SB), in case of disagreement. After preliminary analysis, a quantitative synthesis of the studies was deemed inappropriate given the heterogeneity across the studies and the lack of sufficient comparable interventions and outcomes.

## Results

**Fig. 1:**
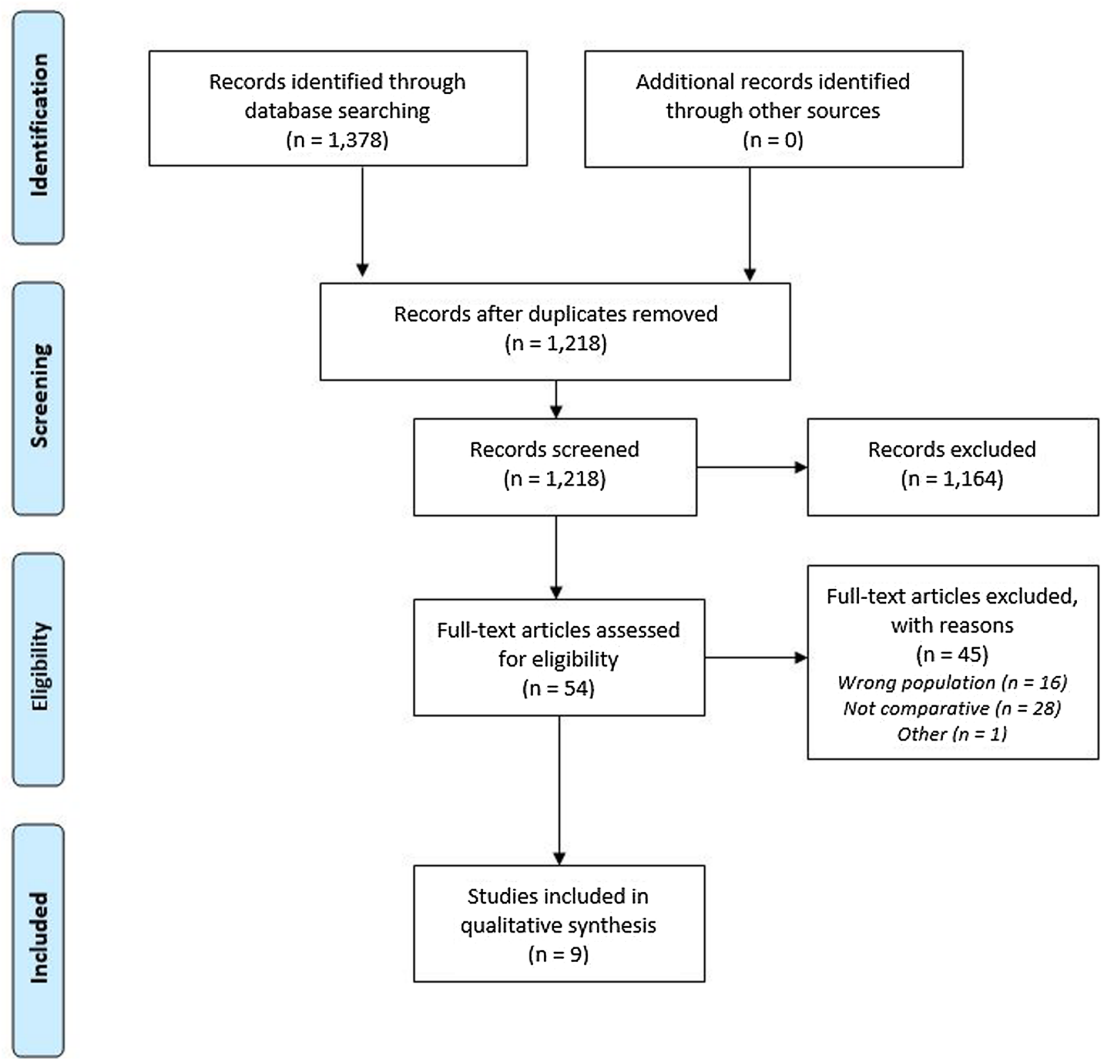
Flow diagram of the study using the PRISMA recommendations [10]

Searches of Scopus, Embase and PubMed databases returned 759, 89, and 342 records, respectively. The PRISMA flow diagram is shown in Fig. 1.

After duplicates were removed, 1218 unique records remained. Screening titles and abstracts eliminated 1164 ineligible records, and full-text articles were retrieved for the remaining 54. Forty-five articles were excluded as they either included the wrong population (*n* = 16), did not report comparative outcomes (*n* = 28), or were conference abstracts superseded by a journal article (*n* = 1) [15, 16]. Nine studies listed in Table 2 met all eligibility criteria [15, 17–24].

**Table 2:** Studies included in the qualitative synthesis

Study	Country	Population				Interventions compared	Outcomes		
		ACS Subclass	Frailty Scale	Age mean (SD) / *Med [IQR]*	Number Frail ^e^		Primary Outcome Measure	Results	
Di Bari et al. [17]^a^	Italy	AMI	Silver Code	82.0 (0.3)^c^	62	PCI vs	One-year mortality	HR = 0.26 (95% CI 0.14–0.48)	HR decreased progressively with increasing silver code scores
				85.0 (0.3) ^c^	116	no PCI			
Alonso et al. [15]^a^	Spain	AMI	SHARE-FI	83.1 (5.1)	58	Invasive strategy vs	One-year Death or MI	41.4%	*p* = 0.078
				87.7 (5.6)	22	conservative strategy		59%	
Nunez et al. [18]^a^	Spain	NSTEACS	Fried Score	78 (7.0)^c,d^	96 ^d^	PCI vs	Long-term all-cause readmission	IRR = 0.6 (95% CI 0.43–0.84) *p* = 0.001	Significant "Frailty status by PCI" interaction (*p* < 0.05)
						no PCI			
Llao et al. [19]^a^	Spain	NSTEACS	FRAIL Scale	86.7 (4.0)^c^	47	Conservative strategy vs	6-month Death, MI or unplanned revascularisation	HR = 1.40 (95% CI 0.72–2.75) *p* = 0.325	Significant "Frailty status by invasive treatment" interaction
				83.6 (3.8)^c^	98	Invasive strategy			
Dodson et al. [20]^a^	U.S	AMI	Study-specific measure	82.2 (8.6) ^d^	3,213	Invasive treatment: frail vs non-frail	In-hospital major bleeding	OR = 1.40 (95% CI 1.24–1.58)	Significant "Frailty status by invasive treatment " interaction (*p* < 0.001)
					3,782	Conservative treatment: frail vs non-frail		OR = 0.96 (95% CI 0.81–1.14)	
Damluji et al. [21]^a^	U.S	AMI	CFI	85.9 (NR)^d^	13,832	PCI vs	In-hospital mortality	OR = 0.59 (95% CI 0.55–0.63)	Significant "Frailty status by PCI" interaction (*p* < 0.001)
					63,413	no PCI			
					12,575	CABG vs		OR = 0.77 (95% CI 0.65–0.93)	Significant "Frailty status by CABG" interaction (*p* < 0.001)
					63,413	no PCI			
Kwok et al. [22]^a^	U.S	ACS	HFRS	80.0 (11^d^	966	PCI vs	In-hospital mortality	16.9%	No additional statistics provided
					NR	Conservative strategy vs		15.0%	
					NR	Angio-MM		12.1%	
Wong, Lee, & El-Jack [23]^b^	NZ	ACS	EFT	87.6 (2.8)	47	PCI vs Medical Management	Long-term mortality	43%	HR = 1.0 (95% CI 0.5–2.0)
				88.9 (NR)	NR			54%	*p* = ns
Fishman et al. [24]^b^	Israel	NSTEMI	NR	*86 [83–90]*c	NR	Invasive treatment vs Conservative treatment	Long-term mortality	HR = 0.52 [95% CI 0.34–0.78]	Non-significant treatment by frailty risk subgroup interaction *p* = ns

*SD* Standard Deviation, *Med* Median, *IQR* Interquartile range, *AMI* Acute Myocardial Infarction, *PCI* Percutaneous Coronary Intervention, *HR* Hazard Ratio, *CI* Confidence Interval

*SHARE-FI* Survey of Health, Ageing and Retirement in Europe Frailty Instrument, *MI* Myocardial Infarction, *NSTEACS* Non-ST-Elevation Acute Myocardial Infarction;

*IRR* Incidence Rate Ratio, *U.S.* United States, *OR* Odds Ratio, *CFI* Claims-Based Frailty Index, *NR* Not Reported, *CABG* Coronary Artery Bypass Grafting, *ACS* Acute Coronary Syndrome

*HFRS* Hospital Frailty Risk Score, *Angio-MM* Angiography without revascularisation, *NZ* New Zealand, *EFT* Essential Frailty Toolset, *ns* Not Significant

^a^Journal article, ^b^Conference abstract, ^c^Total cohort (including non-frail), ^d^No break-down by treatment group, ^e^In the highest risk frailty group

### Characteristics of the included studies

Table 2 summarises the characteristics of included studies. Despite the search allowing for the inclusion of articles published after 1990, all articles were published from 2014 onwards. Data for the studies was generated in only five countries. As shown in Table 2, substantial heterogeneity exists across the attributes of the included studies. Variations in the ACS subtypes included, frailty scales used, coronary interventions compared, and primary outcome measures assessed were of particular interest.

#### Frailty scales and ACS subtypes

Fishman et al. [24] did not clarify the method for assessing frailty in their study. Each of the remaining studies included in this review used a different scale for assessing frailty. Di Bari et al. [17] used the Silver Code [25], a prognostic scoring system for assessing mortality risk rather than frailty risk in patients aged 75 years or older presenting to an emergency department. Dodson et al. [20] used a non-validated, study-specific method for assessing frailty risk.

The remaining studies all used validated frailty scales, but the extent to which these are applicable in the acute coronary setting may differ. Nunez et al. [18] used the Fried score but only assessed frailty at discharge [26]. Damluji et al. [21] used the Claims-based Frailty Index (CFI) [27], derived from a community-based sample, and benchmarked against the Fried score. Alonso et al. [15] used SHARE-FI, which was derived from and validated in a large population-based survey [28]. The Hospital Frailty Risk Score (HFRS) used by Kwok et al. [22] was developed and validated using broadly representative hospitalized cohorts [29]. Wong, Lee, and El-Jack [23] used the Essential Frailty Toolset (EFT) [30, 31], which has been validated in older patients undergoing transcatheter aortic valve implantation and was recently used in a study of older patients who underwent CABG [30]. Llao et al. [19] used the FRAIL scale [32], which has been validated against the Fried score [33], and has been shown to predict mortality risk in older ACS patients [34].

The studies were heterogeneous with respect to the ACS subgroups studied, e.g., Acute Myocardial Infarction (AMI) vs NSTEACS. However, all ACS subtypes were represented across the included studies. STEMI patients were included in six studies [15, 17, 20–23], NSTEMI patients in all nine studies [15, 17–24], and UA in four studies [18, 19, 22, 23].

#### Coronary interventions compared

The studies used four different approaches to defining treatment and control groups. Alonso et al. [15], Dodson et al. [20], Llao et al. [19], and Fishman et al. [24] compared treatment differences between an invasive treatment and conservative treatment. Di Bari et al. [17], Nunez et al. [18], Wong, Lee, & El-Jack [23], and Damluji et al. [21] compared outcomes between patients treated with PCI and patients who were not. The latter group included patients who did not undergo angiography and patients who underwent angiography but were not revascularized (either by PCI or CABG). Damluji et al. [21] also compared outcomes between CABG treatment and treatment without PCI. Finally, Kwok et al. [22] compared the treatment effects of PCI with those following a conservative strategy and with those who received angiography without revascularization (Angio-MM). Kwok et al. [22] is the only study that reported outcomes in thrombolysis-treated patients, but no formal outcome comparison was performed between different treatments.

#### Primary outcome measures

The studies used several primary outcome measures and follow-up durations, as outlined in Table 3.

**Table 3:** Type and timing of primary outcome measures by study

Type of outcome	Primary outcome measure	Timing of primary outcome measurement		
		In-hospital	Medium term^a^	Long term^b^
Safety	Major-bleeding	Dodson et al. [20]		
Efficacy	Mortality	Kwok et al. [22]; Damluji et al. [21]		Di Bari et al. [17]; Wong, Lee, & El-Jack [23]; Fishman et al. [24]s
	Death, MI^c^ or unplanned revascularisation		Llao et al. (2018)	Alonso et al. [15]
	All-cause readmission			Nunez et al. [18]

^a^Six months ^b^ ≥ 1 year ^c^The composite primary outcome measure in Alonso et al. [15] included only death or MI

Dodson et al. [20] explored the effect of frailty and invasive management concerning a critical safety endpoint, in-hospital major bleeding, as defined by the ACTION Registry-GWTG bleeding model [6, 13, 35]. The primary outcome measures used in the remaining studies are generally accepted as measures of efficacy or effectiveness [6, 13, 36]. These included: in-hospital mortality (Kwok et al. [22], Damluji et al. [21]; medium-term (6-month) Death, MI or unplanned revascularization (Llao et al. [19]); long-term (≥ 1 year) mortality (Di Bari et al. [17]), Wong, Lee and El-Jack [23], and Fishman et al. [24]); long-term death or MI (Alonso et al. [15]); and long-term all-cause readmission (Nunez et al. [18]).

### Outcomes of the included studies

#### Studies comparing invasive treatment to conservative treatment

In Dodson et al. [20], the AMI sample comprised 23.8% STEMI and 76.2% NSTEMI. In invasively treated patients, relative to non-frail patients, the risk of in-hospital major-bleeding was increased in those who were frail (Odds Ratio(OR) = 1.33 [95% Confidence Interval (CI) 1.23–1.44]). However, this risk was not increased in frail patients treated conservatively (OR = 1.01 [95% CI 0.86–1.19]). Adjustment for differences in the distributions of baseline confounders was limited to multivariate logistic regression adjustment.

Alonso et al. [15] observed that in frail AMI patients (34% STEMI and 66% NSTEMI), an invasive strategy led to numerically lower rates of 1-year death or MI than a conservative strategy but did not reach statistical significance (41.4% vs 59%; *p* = 0.078). The risk of major bleeding was not significantly increased in the invasive strategy ACS group (invasive [27.6%] vs conservative [40.9%]; *p* = 0.105).

Llao et al. [19] studied 531 patients with NSTEACS (83.8% NSTEMI and 16.2% UA). It was found that whereas a conservative strategy conferred an increased risk (relative to an invasive strategy), for the primary outcome overall (Hazard Ratio (HR) = 2.66 [95% CI 1.71–4.13]; *p* < 0.001), the risk increase was not significant in the frail group (HR = 1.40 [95% CI 0.72–2.75]; *p* = 0.325).

A sample of 2317 patients aged 80 years or older with NSTEMI was studied by Fishman et al. [24]. Following propensity score matching, invasive treatment vs conservative treatment was observed to significantly reduce all-cause mortality risk (HR = 0.61 [95% CI 0.53–0.71]). This effect was consistent across low frailty risk (HR = 0.74 [95% CI 0.58–0.93]), medium frailty risk (HR = 0.65 [95% CI 0.50–0.85] and high frailty risk (HR = 0.52 [95% CI 0.34–0.78] subgroups, with the treatment by frailty risk subgroup interaction *p* value being not significant.

#### Studies comparing PCI to no-PCI

Overall, of the four studies that compared PCI with no PCI in frail older patients, three studies observed a benefit from PCI in terms of mortality risk reduction (in-hospital or longer-to-long-term). In a cohort of patients with AMI (25% STEMI and 75% NSTEMI), Di Bari et al. (2014) observed that relative to no-PCI, PCI reduced 1-year mortality (HR = 0.38 [95% CI 0.27–0.53]; *p* < 0.001). Moreover, the relative benefit of receiving PCI increased with the silver code scores. In the lowest risk stratum (silver code score 0–3) the hazard ratio was 0.48 (95% CI 0.19–1.21; *p* = 0.121), whereas in the highest risk stratum (silver code score > 11), the hazard ratio was 0.26 (95% CI 0.14–0.48; *p* < 0.001).

In a study of older NSTEACS patients (89.6% NSTEMI and 10.4% UA), Nunez et al. [18] found that PCI-treated frail patients had a lower risk of long-term all-cause readmission than frail patients who did not receive PCI (Incidence Rate Ratio = 0.6 [95% CI 0.43–0.84]). The frailty by treatment interaction was significant (*p* = 0.001) but in the opposite direction to that reported by Llao et al. [19] and Dodson et al. [20], i.e., frail patients derived greater benefit from PCI than non-frail patients. No statistical difference in all-cause mortality was observed between PCI and no PCI in frail patients (Incidence Rate Ratio = 0.64 95% CI [0.36–1.12]).

Wong, Lee, and El-Jack (2019) reported that frail patients with ACS (subtype ratios not reported), treated with PCI derived no benefit, relative to medical management, with regards to long-term (2-year) all-cause mortality (43% vs 54%; HR = 1.0 [95% CI 0.5–2.0]; *p* = ns).

Damluji et al. [21] conducted a retrospective cohort study using data for patients with AMI (subtype ratios not reported) from an administrative database. They found that frail patients benefitted from PCI (vs no-PCI) in terms of in-hospital mortality risk reduction (OR = 0.59 [95% CI 0.55–0.63]).

#### Study comparing CABG to no PCI

In the study by Damluji et al. [21], using the same methods described above, CABG reduced the risk of in-hospital mortality relative to no PCI (OR = 0.77 [95% CI = 0.65–0.93]).

#### Study comparing PCI to a conservative strategy

Using data for frail older ACS patients (77.8% NSTEMI, 21.4% STEMI, and 0.8% UA) in a large administrative database, Kwok et al. [22] reported in-hospital mortality rates for a conservative strategy (15%), Angio-MM (12.1%), PCI (16.9%), CABG (12%) and thrombolysis (40%). They noted that while in-hospital mortality rates were consistently lower for PCI than for other interventions in low-risk frailty patients, the risk associated with PCI in frail patients was higher than in frail patients treated with a conservative strategy. However, Angio-MM was associated with the lowest mortality rate of any studied treatment in frail patients. No statistical testing of these differences was reported. The authors also reported event rates for other in-hospital outcomes, including stroke or transient ischaemic attack (CVA/TIA) and bleeding complications. Bleeding complication rates were similar between a conservative strategy, PCI and CABG but higher in Angio-MM. Rates of CVA/TIA were universally high, as was the rate of bleeding complications in thrombolysis treated patients.

### Risk of bias assessment of the included studies

The comprehensiveness of reporting varied within and between the remaining studies. For instance, items such as between-group comparisons in co-interventions were not reported in Di Bari et al. [17], Kwok et al. [22] and Nunez et al. [18]. The risk of bias for Wong, Lee, and El-Jack [23] and Fishman et al. [24] was not systematically evaluated, given that the abstracts did not contain enough information to make a judgement.

This risk of bias for each of the remaining studies was assessed using the McMaster’s CLARITY group Tool for Assessing Risk of Bias in Observational Studies[14], and is shown in Table 4. The risk of bias for each study was high for at least one item in the tool. Concerning differences in between-group co-interventions, all studies were assessed to be at risk of bias. None of the studies presented tables of baseline characteristics demonstrating a balance between the treatment and control groups on these confounders. However, except for Dodson et al. [20], no studies reported regression adjustment for a sufficiently comprehensive set of confounders. In addition, none of the evaluated studies included matching adjustment for differences in baseline confounders.

**Table 4:** Risk of bias assessment of the included studies, using the tool to assess risk of bias in cohort studies [14]

	Di Bari et al. [17]	Alonso et al. [15]	Nunez et al. [18]	Llao et al. [19]	Dodson et al. [20]	Damluji et al. [21]	Kwok et al. [22]
Was selection of exposed and non-exposed cohorts drawn from the same population?	Definitely Yes	Definitely Yes	Probably Yes	Probably Yes	Definitely Yes	Probably Yes	Definitely Yes
Can we be confident in the assessment of exposure?	Definitely No	Definitely Yes	Probably Yes	Definitely Yes	Probably Yes	Probably Yes	Probably No
Can we be confident that the outcome of interest was not present at start of study?	Definitely Yes	Definitely Yes	Probably Yes	Definitely Yes	Definitely Yes	Definitely Yes	Definitely Yes
Did the study match exposed and unexposed for all variables that are associated with the outcome of interest or did the statistical analysis adjust for these prognostic variables?	Probably No	Definitely No	Probably No	Probably No	Probably Yes	Probably No	Probably No
Can we be confident in the assessment of the presence or absence of prognostic factors?	Probably Yes	Probably Yes	Definitely Yes	Definitely Yes	Probably Yes	Probably Yes	Probably Yes
Can we be confident in the assessment of outcome?	Probably Yes	Probably Yes	Probably Yes	Probably Yes	Probably Yes	Definitely Yes	Definitely Yes
Was the follow-up of cohorts adequate?	Probably No	Probably Yes	Probably No	Probably No	Definitely Yes	Probably Yes	Definitely Yes
Were co-interventions similar between groups?	Probably No	Definitely No	Definitely No	Definitely No	Definitely No	Definitely No	Definitely No

## Discussion

To the best of our knowledge, this is the first systematic review comparing health outcomes between coronary interventions in patients aged 75 years or older with ACS. We found few eligible studies despite the broad set of inclusion criteria and limited exclusion. It is interesting to note that all countries in which the data for these studies were generated rank in the top 29 countries globally for per capita health expenditure [37]. However, while between-country disparities in access to high-cost coronary care for frail older patients with acute coronary syndrome may explain some of the geographic concentration of these studies, it may also reflect a global lack of research on this topic. The eligible studies were all observational and at high risk of bias. Notably, adjustment for confounding factors was either limited or not adequately reported in all of them. Except for Fishman et al. [24], which reported limited information about the matching methods employed, the included studies relied exclusively on regression analysis to adjust for imbalances in baseline characteristics and most included few confounders in their analysis. While regression adjustment is a valuable tool when used in conjunction with other methods to reduce confounding, such as matching, it remains prone to significant bias when used alone [38].

The absence of RCT evidence comparing coronary interventions in frail patients aged 75 years or older is consistent with Lee et al. [40] and Konrat et al. [39], who found that older people are underrepresented in RCTs [39, 40]. Encouragingly, the search returned a protocol for an RCT (MOSCA–FRAIL) that is currently underway in which 178 frail NSTEMI patients aged 70 or older have been recruited to test the hypothesis that an invasive strategy reduces major adverse cardiac events relative to a conservative strategy [41, 42].

A key strength of our study is having used a broad search strategy to address the paucity of eligible studies. Having found a relatively small number of studies likely reflects a fundamental gap in the evidence comparing coronary interventions in frail patients aged 75 years or older, rather than it being an artefact of our search strategy. A limitation of our study is that given the broad inclusion criteria, studies of many different designs and outcomes could be eligible for review and, therefore, preclude performing metanalysis. Indeed, substantial heterogeneity was observed between the included studies making it challenging to identify differential treatment effects.

The heterogeneity between the studies in terms of the frailty scores used is particularly noteworthy and may reflect the lack of a fit-for-purpose frailty score that can be used in the acute cardiovascular setting. The Fried score is widely used across a range of clinical settings [18, 26, 27, 43]. The EFT is the only score developed explicitly in a cardiovascular setting, while the HFRS is the only other score developed and validated using hospitalized cohorts [29, 31]. The Fried score, Frail scale and EFT are phenotypic scores derived from the direct assessment of patients, which can be difficult or ill-advised to obtain in the acute setting [44]. The HFRS and the CFI are accumulated deficit scores that can be derived from administrative data, without physical performance tests but do not incorporate information core to frailty, such as the extent to which a patient is sarcopenic [26, 27, 29, 31].

Besides the limitations described above, eight studies showed either a statistically significant or numerical benefit when comparing a more invasive to a less invasive treatment (invasive treatment vs conservative treatment and PCI vs no PCI, respectively). Comparing any invasive treatment during the index hospitalization to conservative treatment mirrors the clinical decision-making process, and guidelines recommend a routine invasive strategy (angiography within 72 h of first medical contact) for intermediate-to-high risk NSTEACS patients [6, 13]. However, no PCI is not an ideal control group for PCI. No PCI includes patients who do not undergo angiography (conservative treatment), and, as the angiographic information invariably influences the PCI treatment decision, only patients who undergo angiography should be included in the control group [6, 22, 45, 46]. Furthermore, whether the control group should include Angio-MM patients or CABG patients should be informed by the pattern of coronary artery disease observed during angiography [6, 22, 45, 46]. This said, the consistency of the findings of the PCI vs no PCI studies with those of the three invasive treatment vs conservative treatment studies, in which the invasive treatment was either statistically superior (Fishman et al. [24]) or numerically superior (Alonso et al. [15] and Llao et al. [19]), may represent a signal that frail patients aged 75 years or older with ACS may benefit from invasive treatment.

The potential signal that invasive treatment may reduce the risk of adverse cardiac events in frail patients aged 75 years or older with ACS is also supported by findings in cohorts that are closely related to frail older ACS patients. Tegn et al. [47] and Malkin, Prakash and Chew. [48] observed that in patients aged 75 years or older with NSTEACS and ACS, respectively, the relative reduction in the risk of adverse cardiac events from an invasive treatment vs conservative treatment increases with age—perhaps only peaking at around 90 years [47, 48]. The MOSCA RCT compared an invasive strategy to a conservative strategy in older, highly comorbid NSTEMI patients and found the risk of adverse cardiac events to be significantly reduced in the invasive strategy group [49]. The findings of Dodson et al. (2019) caution that any benefit in adverse cardiac event risk reduction from an invasive strategy may come at the cost of an increased risk of major bleeding.

Data is lacking with respect to the comparative outcomes between PCI and CABG in frail patients 75 years or older with ACS. Little data is available to warrant conclusions about the relative efficacy of thrombolysis vs other coronary interventions. The mortality rates reported in Kwok et al. [22], combined with related research in patients aged 75 years or older with STEMI, suggest that thrombolysis should be used with caution in frail patients aged 75 years or older [50, 51].

As may be expected due to the relative predominance of NSTEMI vs STEMI in patients aged 75 years or older, all the studies included substantial proportions of NSTEMI patients (range: 66–89.6%). As such, any conclusions drawn from these studies may be more robust for NSTEMI than for STEMI or UA patients.

Limited evidence exists to inform the optimal coronary interventions (i.e., strategies and reperfusion procedures) for frail patients aged 75 years or older with ACS. Drawing conclusions from available observational evidence is limited by the incomplete statistical adjustment required for robust causal analysis. The evidence, such as it is, suggests that there may be a signal for improved outcomes in ACS patients treated invasively vs conservatively. Unfortunately, the accumulation of gold-standard RCT evidence is likely to be hindered by the many challenges associated with conducting RCTs in frail older, acutely unwell patients. In the absence of RCT evidence, observational cohort studies implementing robust methods to achieve confounder balance between treatment and control groups can play an essential role in informing the optimal selection of coronary interventions in frail patients aged 75 years or older with ACS—particularly those with NSTEACS. Retrospective cohorts derived from large data sets with extensive capture of baseline characteristics or prospective registries with disease-and-treatment specific clinical response forms and sufficient power are viable options for cohort studies. The development of a frailty risk score suitable for the acute cardiovascular setting is urgently needed. It should consider the information from which the score will be derived, the feasibility of obtaining the required information, the validity of the score in the hospital-based setting, and the applicability of the score in the context of ischaemic heart diseases.

## Appendix

See Table 5

**Table 5:** Search strings used for each database

Database	Search
Scopus	ALL ( ( "frail" OR "multimorbid" OR "highly comorbid") AND ( "elderly" OR "older" OR "old") AND ( "acs" OR "acute coronary" OR "myocardial infarction" OR "unstable angina" OR "stemi" OR "nstemi" OR "nsteacs" OR "nste-acs" OR "ua") AND ( "pci" OR "percutaneous coronary intervention" OR "angiogra*" OR "invasive management" OR "invasive strategy" OR "medical management" OR "conservative strategy" OR "conservative management" OR "cabg" OR "coronary artery bypass" OR "thromboly*") AND ( "causa*" OR "treatment effect" OR "treatment benefit" OR "treatment outcomes" OR "versus" OR "vs" OR "compar*")) AND PUBYEAR > 1989 AND ( LIMIT-TO ( DOCTYPE, "ar") OR LIMIT-TO ( DOCTYPE, "cp")) AND ( LIMIT-TO ( LANGUAGE, "English"))
Embase	("frail" or "multimorbid" or "highly comorbid").af ("elderly" or "older" or "old").af ("acs" or "acute coronary" or "myocardial infarction" or "unstable angina" or "stemi" or "nstemi" or "nsteacs" or "nste-acs" or "ua").af ("pci" or "percutaneous coronary intervention" or "angiogra*" or "invasive management" or "invasive strategy" or "medical management" or "conservative strategy" or "conservative management" or "cabg" or "coronary artery bypass" or "thromboly*").af ("causa*" or "treatment effect" or "treatment benefit" or "treatment outcomes" or "versus" or "vs" or "compar*").af 1 and 2 and 3 and 4 and 5 limit 6 to (english language and yr = "1990 -Current")
PubMed	((((frail OR multimorbid OR (highly comorbid)) AND (elderly OR older OR old)) AND (acs OR acute coronary OR (myocardial infarction) OR (unstable angina) OR stemi OR nstemi OR nsteacs OR nste-acs OR ua)) AND (pci OR (percutaneous coronary intervention) OR angiogra* OR (invasive management) OR (invasive strategy) OR (medical management) or (conservative strategy) OR (conservative management) OR cabg OR (coronary artery bypass) OR thromboly*)) AND (causa* OR (treatment effect) OR (treatment benefit) OR (treatment outcomes) OR versus OR vs OR compar*) Filters: English, from 1990/1/1 to 2022/5/07
